# Functional skills in *MECP2* duplication syndrome: developmental dynamics and regression

**DOI:** 10.1186/s13023-025-04008-4

**Published:** 2026-01-14

**Authors:** Daniel Ta, Jenny Downs, Gareth Baynam, Peter Richmond, Andrew Wilson, Helen Leonard

**Affiliations:** 1 The Kids Research Institute Australia, University of Western Australia, 6009 Nedlands, WA Australia; 2 Curtin School of Allied Health, Curtin University, Perth, WA Australia; 3 WA Department of Health, Genetic Services of Western Australia, Subiaco, WA 6008 Australia; 4 Western Australian Register of Developmental Anomalies, King Edward Memorial Hospital, PO Box 134, Subiaco, WA 6904 Australia; 5 Rare Care Centre, Perth Children’s Hospital, 15 Hospital Ave, Nedlands, WA 6009 Australia; 6 Perth Children’s Hospital, 15 Hospital Ave, Nedlands, WA 6009 Australia; 7 Discipline of Paediatrics, School of Medicine, University of Western Australia, Perth, UWA 6009 Australia

**Keywords:** *MECP2* duplication syndrome, X-linked, Intellectual disability, Neurodevelopmental, Functional skills, Regression

## Abstract

*MECP2* duplication syndrome (MDS) is an ultrarare, X-linked neurodevelopmental disorder that is poorly understood in terms of its natural history and phenotypic variability. There is limited information on how individuals with MDS acquire, retain or lose fundamental functional skills (gross motor, purposeful hand function and communication) – that of which this study aimed to better characterise in the largest case series to date.

For 160 individuals with MDS (median age 9.06 y, range: 0.57–51.63 y; 84% male), we report that phenotypic penetrance in females can, in some, result in a similar functional skill deficits to males. However, a higher proportion of females acquired gross motor and fine motor skills compared to males. Use of words was the most common parent-reported skill regression (34/90 [38%]) followed by fine motor/hand function (26/90 [29%]), independent walking (25/90 [28%]) and feeding (25/90 [28%]). Additionally, lower proportions of functional ability were present in those with seizures compared to those without. A general trend was also observed for decreasing functional skills with increasing age. Additionally, those with a larger duplication length (1 + Mb) were less likely to be able to acquire independent walking compared with those with less than a 1 + Mb duplication (*p* < 0.001).

This is the first study to comprehensively map the developmental trajectory of functional skills in MDS and provides a seminal baseline for better characterising the natural history of this disorder. Further investigations are required to understand the importance of interventional therapy on the retainment of functional skills.

## Highlights

This is the largest study to date to investigate the acquisition, retention and loss of functional skills in *MECP2* duplication syndrome (MDS).

Females were more likely than males to acquire gross and fine motor skills.

Use of words was the most common parent-reported skill regression.

Those with seizures had lower functional ability than those without seizures.

There is a need for better understanding of the role of interventional therapy for functional skill retention in MDS.

## Introduction

*MECP2* duplication syndrome (MDS; OMIM 30026) is an ultrarare, X-linked neurodevelopmental disorder with a birth prevalence of approximately 1/150,000 liveborn males [1]. Recent efforts to establish a large database of health information for MDS (MDBase) has considerably expanded the understanding of medical comorbidities associated with this disorder [2, 3]. Notably, we found seizures and recurrent lower respiratory tract infections (LRTIs) to be major health issues in MDS – with seizures found in over 60% of individuals with a median onset of 8 years in males and 9.8 years in females. However, there is limited information on the acquisition of functional skills in MDS and any regression of learned skills which has been described in approximately 40% of individuals with MDS [3]. Few studies to date have focussed on profiling this aspect of the natural history of MDS other than two from our group which provided estimates for the likelihood of ascertaining independent sitting and walking as well as the clinical symptoms of seizures, spinal curvature, stereotypic hand movements and speech regression [3, 4]. 

Previous case studies and series have reported on the proportion of individuals that experienced regression of such skills but with limited detail [5]. In our previous review [5], we identified that the regression of gross motor skills was reported in 36/93 (39%) over nine studies, purposeful hand use in 14/67 (21%) over 14 studies and speech/communication skills in 13/25 (52%) of individuals in four studies. A recent study indicated that regression was associated with the onset of epilepsy or progression to treatment-resistant epilepsy in 20/22 (91%) of individuals compared to a spontaneous regression in 5/25 (20%) of those without seizures [6]. Another study has noted that regression was not associated with duplication size [7]. To better understand the regression developmental dynamics in MDS, a stronger foundational understanding of the clinical trajectory is essential.

Here we aim to expand upon previous work by describing the development and loss of functional abilities in MDS using data from the international *MECP2* Duplication Database (MDBase) [2]. 

## Methods

### Data source and study population

MDBase was established in 2020 and collects health-related information from families of individuals with MDS in the form of responses to a comprehensive online questionnaire [2]. The questionnaire and data collection tools were developed in consultation with a consumer reference group of family stakeholders and through review of the literature. The questionnaire captures information on the acquisition, regression and current function for gross motor (relating to sitting, standing, transitions and walking), fine motor (relating to hand function) and communication skills (relating to use of verbal and non-verbal forms of expression). Participant demographics, medical comorbidities including cardiopulmonary health, epilepsy (onset and presence of seizures), gastrointestinal health, ear, nose and throat (ENT) health, musculoskeletal health, kidney/urinary tract health and pain sensitivity are also captured, as explored in our recent publication [3]. 

Since the launch of the MDBase on 13 April 2020 until the census date of 26 June 2023, caregivers of 252 individuals with MDS have been registered. Families of individuals with MDS who previously contributed data towards the International Rett Syndrome Database (InterRett) [4, 8], were first to be invited to register. Further ascertainment was obtained through social media advertising, word-of-mouth via healthcare professionals and families, and at the 2020 and 2022 *MECP2* Duplication Syndrome Conferences. Email contact, phone and/or videoconferencing was initiated and caregivers were invited to complete the online questionnaire and provide genetic reports where possible via upload or by email. Data on the size of the duplication was extracted from genetic reports, where available, and were classed into two groups by length: 0 – <1 Mb and 1 + Mb.

Ethics approval was obtained from the University of Western Australia Human Research Ethics Committee (2019/RA/4/20/5929).

### Gross motor, fine motor and communication skill questionnaire items

Gross motor, fine motor and communication skills were investigated for acquisition, regression and current function. Parents were asked to indicate whether these skills were acquired and the age of their acquisition. Similarly, parents were prompted to recall any partial or complete loss of any previously learned skills, the age at which they regressed and any serious medical issues at the time.

To investigate current gross motor function, a modified version of the Rett Syndrome Gross Motor Scale was used [9]. Originally designed for Rett syndrome, the 15-item gross motor scale encompasses questions on the degree of assistance needed for sitting, standing, transition and walking tasks on a 4-point scale (no assistance, mild assistance, moderate assistance, maximal assistance or unable). Sitting and standing motor skills were coded for those aged 9 months and 1 year or older respectively, whilst transition and walking skills were coded for those aged 1.5 years and 2 years or older to avoid individuals being incorrectly scored on tasks that were too complex for their chronological age.

To investigate current hand function, questions describing the eight levels in the Rett Syndrome Hand Function Scale were administered [10], ranging from being unable to pick up objects to being able to pick up a range of objects with precision. Parents were asked to report how the child achieved grasping activities using large and small objects with yes/no questions.

To investigate current communication skills, questions were derived from the communication matrix [11], a clinical assessment tool suitable for children with severe communication disorders. Parents were asked to select the modes of communication their child usually used e.g., facial expressions, body language, sounds/vocalizations, babble, sign language, communication aids, single words, phrases and/or sentences.

### Data analysis and management

Descriptive statistics were reported as median and range values for continuous variables or proportions and percentages for categorical variables and used to explore the distribution of functional abilities across gender, age group, seizure status and duplication size groups.

Variables describing current gross motor skills were re-categorized as a binary variable (no assistance needed/any assistance or unable). For current hand function skills, the variables were combined: (e.g. using thumb to help pick up small objects or food with precise positioning of the hand before picking up small objects or food). For current communication skills, the following skills were combined (use of facial expressions with body language), (use of sounds/vocalisations with babbling), (use of sign language with communication aids) and (use of single words with phrases and sentences).

**Table 1:** Cohort characteristics of the international *MECP2* duplication database (MDBase) from data collected between April 2020 to June 2023

	Males		Females	Total
**Median age** **(range)**	8.87 y (0.57–51.63 y)		10.18 y (2.31–31.38 y)	9.06 y (0.57–51.63 y)
**Age at census date (years)**	***N***		***N***	***N*** ** (%)**
0 – <6 6 – <12 12 – <18 18+	50 37 26 22		5 12 4 4	55 (33%) 49 (32%) 30 (18%) 26 (17%)
**All age groups (%)**	135 (84%)		25 (16%)	160 (100%)
**Age at genetic diagnosis (years)**	***N***		***N***	***N*** ** (%)**
< 1 1 – <2 2 – <5 5 – <15 15 – <25 25+	31 32 24 26 11 3		5 2 6 7 2 1	36 (24%) 34 (23%) 30 (20%) 33 (22%) 13 (9%) 4 (3%)
**Total**	127		23	150 (100%)
**Size of ** ***MECP2 *** **duplication**	***N***		***N***	***N*** ** (%)**
0–1 Mb	62		11	73 (65%)
1–5 Mb	21		1	22 (19%)
5–10 Mb	5		4	9 (8%)
10 + Mb	9		-	9 (8%)
**Total**	97		16	113 (100%)
**Geographic distribution**		***n/N*** ** (%)**		
Americas^a^		80/160 (50%)		
Europe^b^		51/160 (32%)		
Oceania^c^		24/160 (15%)		
Asia^d^		5/160 (3%)		

^a^USA, Canada and Brazil

^b^UK, Italy, Netherlands, Spain, Poland, Germany, Norway, Denmark, Belgium, France, Finland, Sweden, Switzerland, Andorra, Hungary, Romania and Russia

^c^Australia and New Zealand

^d^Japan, Taiwan, India and United Arab Emirates

**Table 2:** Mapping of independent sitting, independent walking, hand function and use of words across ascertainment, regression and current function domains

	Acquisition		Loss of skill	Current function^*^
	*n*/*N* (%)	Median age (range)	*n*/*N* (%)	*n*/*N* (%)
	**Sitting independently** ^a^			
**Males**	117/133 (88%)	1 y (0.8–5 y)	69/117 (59%)	48/129 (37%)
**Females**	24/25 (96%)	1 y (0.8–3.25 y)	7/23 (30%)	16/24 (67%)
**Total**	142/158 (89%)	1 y (0.8–5 y)	78/142 (55%)	64/154 (42%)
	**Walking independently** ^b^			
**Males**	66/123 (54%)	2.7 y (1.6–7 y)	29/66 (44%)	37/117 (32%)
**Females**	16/24 (67%)	2.8 y (1.7–4.5 y)	3/16 (19%)	13/22 (59%)
**Total**	82/147 (56%)	2.7 y (1.6–7 y)	32/82 (39%)	50/139 (36%)
	**Pincer grasp** ^c^			
**Males**	88/127 (69%)	1.6 y (1.1–2.2 y)	27/88 (31%)	61/129 (47%)
**Females**	18/23 (78%)	1.5 y (1.1–2.3y)	5/18 (28%)	13/23 (52%)
**Total**	106/150 (71%)	1.5 y (1.1–2.3y)	32/106 (30%)	74/154 (48%)
	**Use of single words** ^c^			
**Males**	51/131 (39%)	2.7 y (1.1–6 y)	29/51 (57%)	22/133 (17%)
**Females**	16/24 (67%)	2 y (1.2–7 y)	8/16 (50%)	8/24 (33%)
**Total**	67/155 (43%)	2.5 y (1.1–7 y)	37/67 (55%)	30/157 (19%)

^*^At age of censor date: Males (median age: 8.87 y [range: 0.57–51.63 y]), Females (median age: 10.18 [range: 2.31–31.38 y]);

^a^Restricted to individuals 9 months or older;

^b^Restricted to individuals 1.5 years or older;

^c^Restricted to individuals 1 year or older

Chi-square tests were used to investigate associations between categorical variables. Time-to-event analysis was used to estimate the median age of acquiring independent sitting and walking and pincer grasp and use of word/s. Logistic regression was performed to assess the relationships between functional abilities (current ability to walk (10 steps), use of words, phrases and/or sentences in communication) and gender, age group, seizure status and duplication size with the odds ratio (OR) for each predictor being interpreted as the relative odds of an individual having a higher or lower outcome in the functional ability variable.

## Results

### Study population

From 13 April 2020 to 26 June 2023, the MDBase questionnaire was administered to families of 220 individuals with MDS, of whom 160 (73%) at least partially completed the questionnaire and of these 147/160 (92%) fully completed all questions.

Data were provided for 135 males (84%) and 25 females (16%; Table 1), with 4 male sibling pairs and 18 individuals (15 male, 3 female) who died prior to the census date. The median age at data provision was 8.9 years (range = 0.6–51.6 years) for males and 10.2 years (range = 2.3–31.4 years) for females. At census date, approximately two thirds (65%, 104/160, [87 males, 17 females]) of individuals were aged below 12 years and a third (35%, 56/160 [48 males, 8 females]) above 12 years.

Slightly less than half [47%, *n* = 70) of all individuals were genetically diagnosed with MDS when younger than two years, 20% (*n* = 30) between two and five years, 22% (*n* = 33) between five and 15 years, and 12% (*n* = 17) aged 15 years and above. Genetic reports were available for 137/160 (86%) of individuals (with the length of *MECP2* duplication available for 113/137 (82%) – most (65%, *n* = 73) individuals had a duplication length of 0 – <1 Mb, followed by a fifth (19%, *n* = 22) with a duplication length of 1 – <5 Mb, nine (8%) with a duplication length of 5 – <10 Mb and nine (8%) with a duplication length of 10 + Mb.

Half of individuals were from North and South America (*n* = 80 [50%]), followed by Europe (*n* = 51 [32%]), Oceania (*n* = 24 [15%]), Asia (*n* = 5 [3%]).

### Acquisition and regression of sitting, walking, hand function and use of word/s

**Fig. 1:**
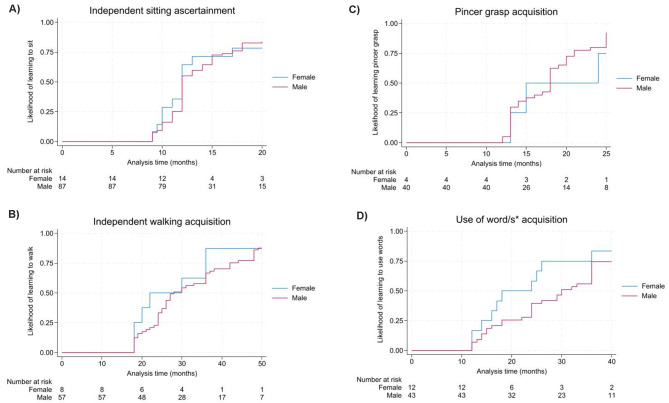
**(A-D)**. Time to event analysis for the acquisition of sitting independently, walking independently, hand function and use of words. **A**) **Sitting**: 50% likelihood of learning to sit by 12 months for females (range: 9–39 months) and 50% likelihood of learning to sit by 12 months for males (range: 9 months – 5 years). Pearson Chi-square *p*-value: 0.84. **B**) **Walking**: 50% likelihood of learning to walk independently by 22 months for females (range: 18 months – 4.5 years) and 30 months for males (range: 18 months – 7 years). Pearson Chi-square *p*-value: 0.52. **C**) **Pincer grasp**: 50% likelihood of learning pincer grasp by 15 months for females (range: 13 months – 26 months) and 18 months for males (range: 12 months – 27 years). Pearson Chi-square *p*-value: 0.50. **D**) **Use of word/s**: 50% likelihood of learning to use words by 18 months for females (range: 12 months – 7 years) and 30 months for males (range: 12 months – 6.5 years). Pearson Chi-square *p*-value: 0.17. ^*^Parent-reported age at which individuals acquired the use of at least one word in their communication

Most (142/158 [89%]) acquired independent sitting; (Table 2) including the majority of (117/133 [88%]) males and all but one female (24/25 [96%]). The median age of acquiring independent sitting was 1 year for both males and females estimated using time-to-event analysis (Fig. 1A). At census date, two-fifths (64/154 [42%]) were able to sit independently and of the 142 individuals that had learned this skill, 78 (55%) experienced regression. Notably, two thirds of males (69/117 [59%]) experienced regression of independent sitting compared to under a third of females (7/23 [30%]).

**Table 3:** Parent-reported skill regression in individuals with MDS by serious medical issue at time of regression and median age of onset

Domain		Proportion of individuals with a parent-reported skill regression	Proportion of individuals with a parent-reported skill regression with:		
			Seizure-related medical issues at time of regression	Respiratory illness-related medical issues^*^ at time of regression	Other-related medical issues^**^ at time of regression
		*n/N* (%)	*n/N* (%)	*n/N* (%)	*n/N* (%)
**Gross motor**	**Sitting**	15/90 (17%)	9/15 (60%)	1/15 (7%)	5/15 (33%)
	**Median age at skill regression (range)**	8 y (3–16 y)	8 y (3–12 y)	16 y	10 y (3–15 y)
	**Standing**	8/90 (9%)	4/8 (50%)	1/8 (13%)	3/8 (38%)
	**Median age at skill regression (range)**	6.7 y (3–31.2 y)	8.7 y (5.5–31.2 y)	8 y	3 y (3–7 y)
	**Unassisted walking**	25/90 (28%)	14/25 (56%)	-	11/25 (44%)
	**Median age at skill regression (range)**	10.5 y (2.6–35 y)	8.8 y (2.6–35)	-	13.5 y (3–25.6 y)
**Fine motor**	**Fine motor/hand function**	26/90 (29%)	17/26 (65%)	3/26 (12%)	6/26 (23%)
	**Median age at skill regression (range)** ^a^	9 y (1.5–22.6 y)	8 y (1.5–22.6 y)	16.4 y (5.8–20.8 y)	9.8 (8.5–10 y)
**Communication**	**Use of words/speech**	34/90 (38%)	10/34 (29%)	3/34 (9%)	22/35 (63%)
	**Median age at skill regression (range)** ^a^	5 y (1.3–22.5 y)	5.3 y (3–22.5 y)	2.5 y (1.5–3 y)	4.2 y (1.3–12 y)
**Activities of daily living (ADL)**	**Feeding**	25/90 (28%)	14/25 (56%)	4/25 (16%)	7/25 (28%)
	**Median age at skill regression (range)**	11 y (1.5–40 y)	12.8 y (3–40 y)	5.8 y (1.5–16.2 y)	10.5 y (5.8–18 y)
	**Toileting**	6/90 (7%)	3/6 (50%)	1/6 (17%)	2/6 (33%)
	**Median age at skill regression (range)**	6 y (2.5–30.8 y)	6 y (4.5–30.8 y)	2.5 y	10 y

^*^Reported medical issues include recurrent pneumonia and aspiration;

^**^Reported medical issues include constipation, falls, hospitalisation, infection, leg spasticity, leukaemia, surgery, scoliosis and unspecified/complex comorbidity profile;

^a^Restricted to individuals 1 year or older

**Table 4:** Current gross motor skills requiring no assistance in individuals with MDS by gender, age group and presence of seizures

		Sitting		Standing		Transitions				Walking					
		Sitting on a stool^a^	χ^2^; *p*	Standing 20 seconds^b^	χ^2^; *p*	Sitting to standing^b^	χ^2^; *p*	Floor to standing^b^	χ^2^; *p*	Walking 10 steps^c^	χ^2^; *p*	Walking up or down a slope^c^	χ^2^; *p*	Running^d^	χ^2^; *p*
	**Total** ***n/N*** **(%)**	64/153 (42%)	-	52/150 (35%)	-	49/152 (32%)	**-**	40/151 (26%)	-	50/139 (36%)	**-**	23/143 (16%)	-	22/142 (15%)	-
**Gender**	**Males** ***n/N*** **(%)**	48/129 (37%)	7.22; **0.007**	39/127 (31%)	5.73; **0.017**	36/128 (28%)	6.27; **0.012**	29/127 (23%)	5.48; **0.019**	37/117 (32%)	6.07; **0.014**	12/119 (10%)	18.91; **< 0.001**	11/118 (9%)	20.31; **< 0.001**
	**Females** ***n/N*** **(%)**	16/24 (67%)		13/23 (57%)		13/24 (54%)		11/24 (46%)		13/22 (59%)		11/24 (46%)		11/24 (46%)	
**Age group**	**< 6 years** ***n/N*** **(%)**	24/51 (47%)	2.05; 0.562	15/49 (31%)	4.62; 0.202	14/50 (28%)	2.35; 0.503	14/50 (28%)	4.35; 0.226	14/40 (35%)	2.04; 0.564	5/41 (12%)	5.37; 0.147	5/40 (12%)	2.29; 0.514
	**6 – < 12 years** ***n/N*** **(%)**	21/47 (45%)		19/46 (41%)		17/47 (36%)		12/46 (26%)		19/45 (42%)		11/47 (23%)		9/47 (19%)	
	**12 – <18 years** ***n/N*** **(%)**	11/30 (37%)		13/30 (43%)		12/30 (40%)		11/30 (37%)		11/30 (37%)		6/30 (20%)		6/30 (20%)	
	**18 + years** ***n/N*** **(%)**	8/25 (32%)		5/25 (20%)		6/25 (24%)		3/25 (12%)		6/24 (25%)		1/25 (4%)		2/25 (8%)	
**Seizure status**	**Seizures (Y)** ***n/N*** **(%)**	27/89 (30%)	11.55; **0.001**	19/88 (22%)	17.47; **< 0.001**	18/89 (20%)	13.95; **< 0.001**	14/89 (16%)	12.48; **< 0.001**	18/85 (21%)	22.68; **< 0.001**	5/88 (6%)	19.38; **< 0.001**	7/87 (8%)	10.2; **0.001**
	**Seizures (N)** ***n/N*** **(%)**	36/62 (58%)		33/60 (55%)		30/61 (49%)		25/60 (42%)		32/52 (62%)		18/53 (34%)		15/53 (28%)	

^a^Restricted to individuals 9 months or older;

^b^Restricted to individuals 1 year or older;

^c^Restricted to individuals 1.5 years or older;

^d^Restricted to individuals 2 years or older

**Table 5:** Current hand function skills in individuals with MDS by gender, age group and presence of seizures as a comorbidity

		Pressing a switch (e.g., to operate a device)^a^	χ^2^; *p*	Grasping, lifting and holding large object for at least 2 seconds^a^	χ^2^; *p*	Raking fingers when picking up small objects or food^a^	χ^2^; *p*	Using thumb or precise positioning of hand before picking up small objects or food^a^	χ^2^; *p*
	**Total** ***n/N*** **(%)**	108/153 (71%)	-	122/154 (79%)	-	117/151 (77%)	-	74/154 (48%)	-
**Gender**	**Males** ***n/N*** **(%)**	86/128 (67%)	4.36; **0.037**	99/129 (77%)	2.96; 0.085	97/128 (76%)	1.40; 0.237	61/129 (47%)	0.19; 0.666
	**Females** ***n/N*** **(%)**	22/25 (88%)		23/25 (92%)		20/23 (87%)		13/25 (52%)	
**Age group**	**< 6 years** ***n/N*** **(%)**	28/51 (55%)	10.70; **0.013**	44/51 (86%)	11.33; **0.010**	44/50 (88%)	19.06; < **0.001**	28/51 (55%)	4.83; 0.184
	**6 – < 12** **years** ***n/N*** **(%)**	39/46 (85%)		41/47 (88%)		38/45 (84%)		25/47 (53%)	
	**12 – <18** **years** ***n/N*** **(%)**	22/30 (73%)		22/30 (73%)		23/30 (77%)		13/30 (43%)	
	**18+** **years** ***n/N*** **(%)**	19/26 (73%)		15/26 (58%)		12/26 (46%)		8/26 (31%)	
**Seizure status**	**Seizures (Y)** ***n/N*** **(%)**	61/89 (69%)	1.30; 0.254	62/90 (69%)	13.12; < **0.001**	59/89 (66%)	14.88; < **0.001**	33/90 (37%)	10.84; **0.001**
	**Seizures (N)** ***n/N*** **(%)**	47/61 (77%)		57/61 (93%)		56/60 (93%)		39/61 (64%)	

^a^Restricted to individuals 1 year or older

Just over half (82/147 [56%] learned to walk independently (Table 2), though just over a half (66/123 [54%]) of males but two thirds (16/24 [67%]) of females acquired this skill. The median age of acquiring independent walking was 28 months for males but earlier at 22 months for females (Fig. 1B). At census date, just over a third (50/139 [36%]) were reported to be able to walk independently. Therefore, 32/82 (39%) individuals experienced regression of this skill. Nearly half (19/66 [44%]) of males experienced regression of independent walking compared to only 3/16 (19%) of females.

For hand function, just over two-thirds 106/150 [71%] learned to use a pincer grasp although fewer (88/127 [69%]) males than females (18/23 [78%]) acquired this skill (Table 2). The median age of acquiring a pincer grasp was later for males (18 months) than females (15 months; Fig. 1C). At census date, approximately half (74/154 [48%]) were still able to use a pincer grasp. As such, approximately a third (32/106 [30%]) experienced regression in this skill with similar proportions for males (27/88 [31%]) and females (5/18 [28%]).

Smaller proportions of individuals learned to use single words, with just over two-fifths (67/155 [43%]) acquiring this skill (Table 2). Two-thirds (16/24 [67%]) of females learned to use single words compared to just under two-fifths (51/131 [39%]) of males. The median age of acquiring use of single words was 30 months for males and 18 months for females (Fig. 1D). By census date, only a fifth (30/157 [19%]) were reported to be able to use single words and as such more than half (37/67 [55%]) of individuals had regressed in this skill. Just over half (29/51 [57%]) of males experienced a loss of ability to use single words and similarly a half (8/16 [50%]) for females.

### Parent-reported developmental skill regression

Parents of 90 children and adults provided specific details on the type of regression experienced (Table 3). Lost skills were categorized as gross motor, fine motor, communication or activities of daily living (ADL) skills (Table 3). Regression in independent walking was reported by parents for 25 individuals of whom 14 reported seizure-related issues at time of this regression. Regression in use of words/speech was reported by parents for 34 individuals at a median age of 5 years (range = 1.3–22.5 years). Regression of fine motor/hand function was reported by parents of 26 individuals with a median age of skill regression at 9 years (range = 1.5–22.6 years). Seizures were the most associated medical issue identified by parents in 17 at the time of this regression. A small number (*n* = 25) of individuals were also reported by parents to experience regression in feeding abilities at a median age of 11 years (range = 1.5–40 years) with seizures again being the most associated medical issue occurring in 14.

### Current functioning – Gross motor skills

Less than half (64/153 [42%]) were able to sit on a stool unassisted although this was reported in two-thirds (16/24 [67%]) of females compared to just over a third (48/129 [37%]) of males (Table 4). With increasing age, fewer individuals were able to perform this skill, decreasing from 24/51 (47%) individuals aged younger than 6 years to 8/25 (32%) in those aged 18 years and older. Less than a third (30%, 27/89) with seizures were able to sit without assistance compared to just under three-fifths (58%, 36/62) of those without seizures.

Approximately one third (49/152 [32%]) could transition from sitting to standing. Slightly more than approximately a quarter (14/50 [28%]) of those aged less than 6 years to a third (17/47 [36%]) of those aged between 6 and 12 years. Under half (12/30 [40%]) of those aged between 12 and 18 years could achieve this ability, but this was only reported for 6/25 (24%) of individuals aged 18 years and above. Approximately a fifth (18/90 [20%]) of those with seizures were able to transition from sitting to standing compared to those without (33/60 [55%]).

The ability to walk unassisted at least 10 steps was reported for a third (50/139 [36%]), with over half (59%, 13/22) of females being able to walk compared to a third (37/117 [32%) of males. Less than half were able to walk without assistance with only a quarter (6/24 [25%]) of those aged 18 years and above. Whilst more than half (32/52 [62%]) of those without seizures could perform this task, only a fifth (18/85 [21%]) of those with seizures could.

### Current functioning – Hand function

The ability to rake fingers to pick up small objects or food was reported for approximately three-quarters (117/151 [77%], Table 5). Less males (97/128 [76%]) than females (20/23 [87%]) were able to do this. The proportion of individuals able to rake also reduced with increasing age (*p* < 0.001).

Approximately half (74/154 [48%]) were able to pick up small objects using the thumb or precise position of the hand to pick up small objects. Interestingly, this was similar for males (61/129 [47%]) and females (13/25 [52%]). Just over half of those aged less than 12 years (51/98 [52%]) were able to perform this hand function compared to less than a half (13/30 [43%]) of those aged 12 to 18 years and a third (8/26 [31%]) in those aged above 18 years.

### Current functioning – Communication skills

Less than half (66/152 [43%]) used sounds/vocalisations and babble with more males (61/130 [47%]) than females (5/22 [21%]) using this as part of their communication form (*p* = 0.034; Table 6). This proportion decreased with each increasing age group (*p* = 0.026).

More than half (86/150 [57%]) were reported to use either sign language and/or communication aids and this was similar in proportion between males (73/128 [57%]) and females (13/22 [59%]; *p* = 0.857). Those aged between 6 and 12 years utilised this communication style the most (34/45 [76%]) compared to those younger: aged less than 6 years (24/50 [48%]) and those older: aged between 12 and 18 years (17/30 [57%]) and aged 18 years and older (11/25 [44%]; *p* = 0.021).

Only a quarter (37/150 [25%]) were able to utilise single words, phrases and sentences to communicate, with this being much more common in females (14/22 [64%]) than males (23/128 [18%]. Those aged between 6 and 12 years were reported to use words in communication (15/45 [33%]) more than other age groups (*p* = 0.384; Table 6).

**Table 6:** Current communication skills in individuals with MDS by gender and age group.^*^

		Facial expressions and body language^a^	χ^2^; *p*	Sounds/ vocalisations and babble^b^	χ^2^; *p*	Sign language and communication aids^c^	χ^2^; *p*	Single words, phrases and/or sentences^c^	χ^2^; *p*
	**Total** ***n/N*** **(%)**	140/152 (92%)	-	66/152 (43%)	-	86/150 (57%)	-	37/150 (25%)	-
**Gender**	**Males** ***n/N*** **(%)**	121/130 (93%)	1.17; 0.280	61/130 (47%)	4.48; **0.034**	73/128 (57%)	0.03; 0.857	23/128 (18%)	21.07; **< 0.001**
	**Females** ***n/N*** **(%)**	19/22 (86%)		5/22 (21%)		13/22 (59%)		14/22 (64%)	
**Age group**	**< 6 years** ***n/N*** **(%)**	49/52 (94%)	0.57; 0.904	28/52 (54%)	9.23; **0.026**	24/50 (48%)	9.71; **0.021**	11/50 (22%)	3.05; 0.384
	**6 – < 12 years** ***n/N*** **(%)**	41/45 (91%)		23/45 (51%)		34/45 (76%)		15/45 (33%)	
	**12 – <18 years** ***n/N*** **(%)**	27/30 (90%)		8/30 (27%)		17/30 (57%)		7/30 (23%)	
	**18 + years** ***n/N*** **(%)**	23/25 (92%)		7/25 (28%)		11/25 (44%)		4/25 (16%)	

^*^Individuals may be able to use multiple forms of communication and these reported skills are not indicative of the highest level of communication but inclusive of all forms currently being utilised;

^a^Restricted to individuals 2 months or older;

^b^Restricted to individuals 4 months or older;

^c^Restricted to individuals 1 year or older

### Relationships between gender, age, seizures, duplication length and current functional abilities

Univariate and multivariate analyses for the ability to walk unassisted for 10 steps and using words, phrases and/or sentences are shown in Table 7.

**Table 7:** Univariate and multivariate relationships between gender, seizure age group, presence of seizures and duplication size and current ability to walk and communicate through words

		Univariate models		Multivariate models	
	*N* (%)	OR (95% CI)	*p*	OR (95% CI)	*p*
**Walking 10 steps** ^a^					
**Male**	37 (32%)	Baseline		Baseline^*^	
**Female**	13 (59%)	3.12 (1.23, 7.96)	**0.016**	6.11 (1.44, 25.97)	**0.014**
**1.5–12 yrs**	34 (39%)	Baseline		Baseline^*^	
**12 + yrs**	16 (30%)	0.63 (0.31, 1.31)	0.212	1.52 (0.04, 5.97)	0.551
**Seizures (Y)**	18 (21%)	Baseline		Baseline^*^	
**Seizures (N)**	32 (60%)	5.96 (2.78, 12.78)	**< 0.001**	8.11 (2.45, 26.79)	**0.001**
**0-<1 Mb duplication**	32 (50%)	Baseline		Baseline^*^	
**1 + Mb duplication**	5 (14%)	0.17 (0.06, 0.48)	**< 0.001**	0.19 (0.06, 0.66)	**0.009**
**Use of words**,** phrases and/or sentences**^b^					
**Male**	24(18%)	Baseline		Baseline^*^	
**Female**	14 (58%)	6.69 (2.65, 16.93)	**< 0.001**	10.70 (3.07, 37.30)	**< 0.001**
**1–12 yrs**	27 (26%)	Baseline		Baseline^*^	
**12 + yrs**	11 (19%)	0.71 (0.32, 1.56)	0.384	0.31 (0.08, 1.13)	0.076
**Seizures (Y)**	20 (22%)	Baseline		Baseline^*^	
**Seizures (N)**	17 (27%)	1.26 (0.59, 2.69)	0.547	0.75 (0.27, 2.08)	0.586
**0-<1 Mb duplication**	21 (29%)	Baseline		Baseline^*^	
**1 + Mb duplication**	9 (23%)	0.73 (0.29, 1.80)	0.487	0.64 (0.22, 1.83)	0.402

^*^Adjusted for presence of gender, age group, presence of seizures and duplication length

^a^Restricted to individuals 1.5 years or older, *n* = 153;

^b^Restricted to individuals 1 year or older, *n* = 161;

Unsurprisingly, females were much more likely to perform these skills, with three times the odds (OR 3.12, 95% CI 1.23, 7.96) of walking and six times the odds (OR 6.69, 95% CI 2.65, 16.93) of using words, phrases and/or sentences. The odds increased further when adjusted for gender, age group, presence of seizures and duplication length for walking (OR 6.11, 95% CI 1.44, 25.97) and use of words (OR 10.70, 95% CI 3.07, 37.30) respectively.

Those without seizures were 5.96 times (95% CI 2.78, 12.78) as likely to be able to walk compared to those with seizures using univariate analysis – the odds increasing further when adjusting for the aforementioned factors (OR 8.11, 95% CI 2.45, 26.79). Similarly using univariate analysis for those with a larger duplication size of 1 Mb and above, individuals were approximately 80% less likely to be able to walk (OR 0.17, 95% CI 0.06, 0.48) and use words (OR 0.19, 95% CI 0,06, 0.66) when adjusted in multivariate analysis, compared to those with a smaller duplication size.

There was no apparent relationship between age groups of 1.5 to 12 years and those older than 12 years with walking – nor age group, presence of seizures and duplication size for using words, phrases and/or sentences.

## Discussion

This current study features the largest dataset, 135 males and 25 females, with MDS to date and is the first to provide an in-depth exploration of the trajectory of functional skills in this disorder. Expanding greatly upon previous studies [1, 4, 12, 13], we confirm that females with MDS have greater functional abilities in general. However, we acknowledge that the female phenotype can extend beyond this milder presentation that is often considered as the upper limit of severity [14, 15], as we identified some females with functional skill deficits similar to that of males. Use of words was the most commonly parent-reported skill regression. A general trend of decreasing presence of functional skills with increasing age was observed across all three domains explored in this study: gross motor skills, purposeful hand use and communication skills – as well as a lower proportion of such skills present in those with seizures compared to those without. Finally, we report that a larger duplication length (1 + Mb) was associated with a decreased odds of independent walking.

We found a higher proportion of males acquiring independent walking (66/123 [54%]) in our study than in a recent Japanese study (9/23 [39%]) [13]. Yet our proportion was still lower than the 79% (44/56) reported in a French study [12]. Comparing age of independent walking acquisition amongst males, our study reported a median age of 2.9 years (range: 1.6 y – 24 years) which was earlier than in the Japanese study with a median age of 5 years (range: 1.5–8 years) [13] – meanwhile the French study reported a mean age of 3.6 years (range: 1.8–5.4 years). Interestingly, the 50% likelihood of sitting and pincer grasp acquisition was at a similar age for males and females in our study, which supports the notion that females may have a similar degree of developmental delay to males in some areas. However, it should be noted that at censor date, a third (8/24 [33%]) of females were still able to use single words compared to only 17% (22/133) of males, highlighting the heterogeneity of the female phenotype. Females also had an earlier likelihood of achieving independent walking and use of word/s, although this difference was not statistically significant. Notably for current communication skills (Table 6), a greater proportion of females communicated through single words, phrases or sentences in contrast to males who were more likely to use sounds/vocalisations and babble.

Our current study is the most comprehensive investigation of specific functional skill loss, succeeded by only one prior brief report on 17 boys in which eight exhibited regression in language skills, four in hand use and seven in motor skills (posture, gait, coordination) [7]. In our study, the number of individuals that experienced skill regression (loss of independent sitting, independent walking, pincer grasp and use of words) was deduced from the difference between the number of individuals who acquired the skill and those for whom the skill was retained at data collection (Table 2). While just over a half of individuals lost the ability to sit independently from acquisition to censor date, such a regression was only reported in a specific regression question by fewer parents. This difference is quite likely due to recall error and a gradual loss of skills may have potentially not been identified by some parents as an episode of regression. As such, there may be other instances of skill regression that have not been fully reported by parents in our study (Table 2). Of note, the 39% (32/82) of individuals observed to experience regression in independent walking is a little greater than that reported in the French study (7/24 [29%]) [12]. Additionally, we found that seizure-related issues were commonly associated with skill regression which is in line with previous studies [6, 7, 12]. It is important to consider the impact of seizure control and side effects of antiepileptic drugs on the development/regression of functional skills in future studies.

Apart from the association with seizure onset, the aetiology and mechanism of regression in MDS remains unknown. Interestingly, one novel study found that indviduals with MDS who experienced regression had abnormal diurnal cortisol rhythms that remained at flatter levels throughout the day [16]. In comparison, those who did not experience regression had a typical cortisol response of higher levels in the morning with linear decreases throughout the day [16]. Further studies will be required to better understand the underlying mechanism causing regression in MDS and the value of interventional therapies for recovering functioning. It is notable that in the brief report on 17 males [7], two individuals with language regression alone were able to recover such skills with intensive speech therapy. Furthermore, a recent case study reported on the use of physiotherapy on a six-year old male with MDS which resulted in improvement of gross motor function in domains of lying, rolling, sitting, crawling, kneeling and standing after 12 weeks of treatment [17]. The utility of physical therapy could be evaluated in the context of skill recovery after regression in MDS.

We found those with a larger duplication size (1 + Mb) were less likely to be able to walk independently. This supports previous findings that larger duplication size was correlated with greater phenotypic severity in 48 individuals (43 male, 5 female) with MDS as demonstrated by higher severity scores using the Clinical Severity Scale (CSS) and Motor Behavioural Assessment Scale (MBA) [18]. Additionally, males had significantly higher CSS and MBA scores than females.

We acknowledge that the Rett Syndrome Gross Motor Scale and Rett Syndrome Hand Function Scale, as used in this study, were not originally designed for MDS. However, no scales have been developed specifically for MDS, and the current scales are generally applicable for those with severe functional impairments. Moreover, another study on individuals with MDS has also utilised Rett syndrome-based scales [18]. For the future potential design of such MDS-specific assessment or severity scales, it is important to thoroughly understand the developmental trajectory of functional skills in this disorder for which this current study provides an important baseline. Additionally, parent-reported health-data collected in this study might be subject to recall error and as such, caregivers have been asked to refer to medical records where possible. We also acknowledge that our grouping of age and duplication size may have differed from previous studies [6, 19]. We coded data into broad categories for the regression models and believe that we have captured meaningful variability in the MDS disorder. Further analyses could potentially combine these data on developmental skills with our data on comorbidities in MDS [3], to collectively contribute towards the development of outcome measures for clinical trials, which are of utmost importance for families.

In summary, this study provides a comprehensive overview of functional skills in males and females with MDS adding further granularity to the understanding of the natural history of gross and fine motor and communication skills this disorder. We highlight the associations between gender, age and presence of seizures and these skills and additionally the impact of duplication size on the likelihood of being able to walk independently. The use of our international registry, MDBase, has provided a better depiction of the phenotypic variability, milestone estimates and developmental dynamics of individuals with this disorder through the largest dataset to date. We believe this study will serve as a baseline for future longitudinal studies and contribute to the creation of a severity scale for MDS.

## Data Availability

The datasets used and/or analysed during the current study are available from the corresponding author on reasonable request.
